# HumanMetagenomeDB: a public repository of curated and standardized metadata for human metagenomes

**DOI:** 10.1093/nar/gkaa1031

**Published:** 2020-11-22

**Authors:** Jonas Coelho Kasmanas, Alexander Bartholomäus, Felipe Borim Corrêa, Tamara Tal, Nico Jehmlich, Gunda Herberth, Martin von Bergen, Peter F Stadler, André Carlos Ponce de Leon Ferreira de Carvalho, Ulisses Nunes da Rocha

**Affiliations:** Institute of Mathematics and Computer Sciences, University of São Paulo, São Carlos, Brazil; Department of Environmental Microbiology, Helmholtz Centre for Environmental Research – UFZ GmbH, Leipzig, Saxony 04318, Germany; Department of Computer Science and Interdisciplinary Center of Bioinformatics, University of Leipzig, Leipzig, Saxony 04107, Germany; GFZ German Research Centre for Geosciences, Section 3.7 Geomicrobiology, Telegrafenberg, 14473 Potsdam, Germany; Department of Environmental Microbiology, Helmholtz Centre for Environmental Research – UFZ GmbH, Leipzig, Saxony 04318, Germany; Department of Computer Science and Interdisciplinary Center of Bioinformatics, University of Leipzig, Leipzig, Saxony 04107, Germany; Department of Bioanalytical Ecotoxicology, Helmholtz Centre for Environmental Research – UFZ GmbH, Leipzig, Saxony 04318, Germany; Department of Molecular Systems Biology, Helmholtz Centre for Environmental Research – UFZ GmbH, Leipzig, Saxony 04318, Germany; Department of Environmental Immunology, Helmholtz Centre for Environmental Research – UFZ GmbH, Leipzig, Saxony 04318, Germany; Department of Molecular Systems Biology, Helmholtz Centre for Environmental Research – UFZ GmbH, Leipzig, Saxony 04318, Germany; Institute of Biochemistry, Faculty of Life Sciences, University of Leipzig, Leipzig, Saxony 04107, Germany; Department of Computer Science and Interdisciplinary Center of Bioinformatics, University of Leipzig, Leipzig, Saxony 04107, Germany; Institute of Mathematics and Computer Sciences, University of São Paulo, São Carlos, Brazil; Department of Environmental Microbiology, Helmholtz Centre for Environmental Research – UFZ GmbH, Leipzig, Saxony 04318, Germany

## Abstract

Metagenomics became a standard strategy to comprehend the functional potential of microbial communities, including the human microbiome. Currently, the number of metagenomes in public repositories is increasing exponentially. The Sequence Read Archive (SRA) and the MG-RAST are the two main repositories for metagenomic data. These databases allow scientists to reanalyze samples and explore new hypotheses. However, mining samples from them can be a limiting factor, since the metadata available in these repositories is often misannotated, misleading, and decentralized, creating an overly complex environment for sample reanalysis. The main goal of the HumanMetagenomeDB is to simplify the identification and use of public human metagenomes of interest. HumanMetagenomeDB version 1.0 contains metadata of 69 822 metagenomes. We standardized 203 attributes, based on standardized ontologies, describing host characteristics (e.g. sex, age and body mass index), diagnosis information (e.g. cancer, Crohn's disease and Parkinson), location (e.g. country, longitude and latitude), sampling site (e.g. gut, lung and skin) and sequencing attributes (e.g. sequencing platform, average length and sequence quality). Further, HumanMetagenomeDB version 1.0 metagenomes encompass 58 countries, 9 main sample sites (i.e. body parts), 58 diagnoses and multiple ages, ranging from just born to 91 years old. The HumanMetagenomeDB is publicly available at https://webapp.ufz.de/hmgdb/.

## INTRODUCTION

Metagenomics is the study of the genetic potential of a multispecies assemblage. Its most frequent application is to define all genomes of a microbial community in a culture-independent approach (1). In the past 20 years, high-throughput sequencing prices diminished to a point that metagenomics data increases exponentially (2). Therefore, metagenomes encompass an overwhelming volume of complex data, presenting challenges not only for storage but also for metadata annotation and curation (3). Some repositories provide permanent storage for DNA sequencing. The major database is the Sequence Read Archive (SRA) (4), which is part of the International Nucleotide Sequence Database Collaboration (5), along with the European Nucleotide Archive (ENA) (6) and the DNA Data Bank of Japan (7). Other notable databases are the MG-RAST (8), the EBI Metagenomics, or MGnify (9), gcMeta (10), MSE (11) and Qiita (10).

The availability of these databases allows scientists to reanalyze samples from individual studies or perform meta-analyses across studies to explore new hypotheses. Besides, reanalysis can be justified by the presence of novel methods and bioinformatic techniques, leading to novel discovery and insights (13). For instance, a recent study (14) analyzed eight different fecal shotgun metagenomic studies of colorectal cancer and identified twenty-nine species that were significantly enriched in colorectum cancer metagenomes. This meta-analysis, therefore, yielded colorectal-microbiome species associations with higher certainty than any single study.

The process of mining the samples of interest, however, can be a limiting factor in a meta-analysis. Metadata available in repositories, such as the SRA, are not standardized, creating an overly complex environment for sample reanalysis. As a result, the data is underutilized (15), which is not in the public interest. The difficulty associated with properly accessing metagenome data has led to initiatives such as the Genomic Standards Consortium (16), and the BioProject and BioSample project (17), which defined the minimum necessary information about a metagenomic sample (18). The Genomes OnLine database (GOLD) (19) have an important role on making genome and metagenome sequence data accompanied with its accurate metadata. However, the filtering process of the samples based on more detailed metadata needs further optimization. To address this problem, domain-specific standardization efforts have started to coalesce. For example, the TerrestrialMetagenomeDB release 1.1 contains standardized metadata of almost 20 thousand terrestrial metagenomes (20). In the case of human metagenomic samples, an initiative that improved metadata integration was the Human Microbiome Project (21). In July 2020, the Human Microbiome Project data portal comprised 31 596 samples, ranging from 18 studies, with a file volume of 48.54 TB of data (https://portal.hmpdacc.org/). More recently, the works from Bernstein *et al.* (22) and Pasoli *et al.* (23) curated and standardized metadata from human-specific samples. Although the work from Pasoli *et al.* offers an impressive number of attributes, it is only available via Bioconductor (24) and is stored as an ExpressionSet object in an R environment. This may create overly complex conditions for non-bioinformaticians to identify and select samples. In contrast, the work from Bernstein et al. has a more easily accessible interface with considerable progress on metadata standardization of the SRAdb human samples. However, it is not metagenomic specific, which hinders the task of selecting true metagenome samples. Additionally, the search functionality of both databases is not optimized to help to answer biologically relevant questions. As one example, it is difficult to select samples based on age or body mass indexes.

To address these limitations, we created the HumanMetagenomeDB (HMgDB), which integrates metadata from metagenomes recovered in multiple studies and body-sites in a single standardized database with curated metadata. In this novel database, users can easily filter the data for samples of interest, including standardized attributes describing host characteristics (e.g. sex, age and body mass index), diagnosis information (e.g. cancer, Crohn's disease and Parkinson), location (e.g. country of origin, longitude and latitude), sampling site (e.g. gut, lung and skin) and sequencing attributes (e.g. sequencing platform, average length and sequence quality). The HMgDB will, therefore, enable scientists to easily find metagenomes of interest and, as a result, lead to better and more accurate meta-analyses.

Overall, the HMgDB integrates metadata relevant to its biological information and metadata describing the technical aspects of the sequencing information. The database is not meant to replace recent efforts of the BioSamples database to standardized and curate data (25). The HMgDB was instead created to promote exploratory possibilities of human metagenomes in a user-friendly interface, encouraging a broader comparison of publicly available data. The HMgDB combines the metadata present in the SRA and MG-RAST repositories, providing manually curated and standardized metadata that could be useful in further meta-analysis studies.

## MATERIALS AND METHODS

### Database construction

**Figure 1.**
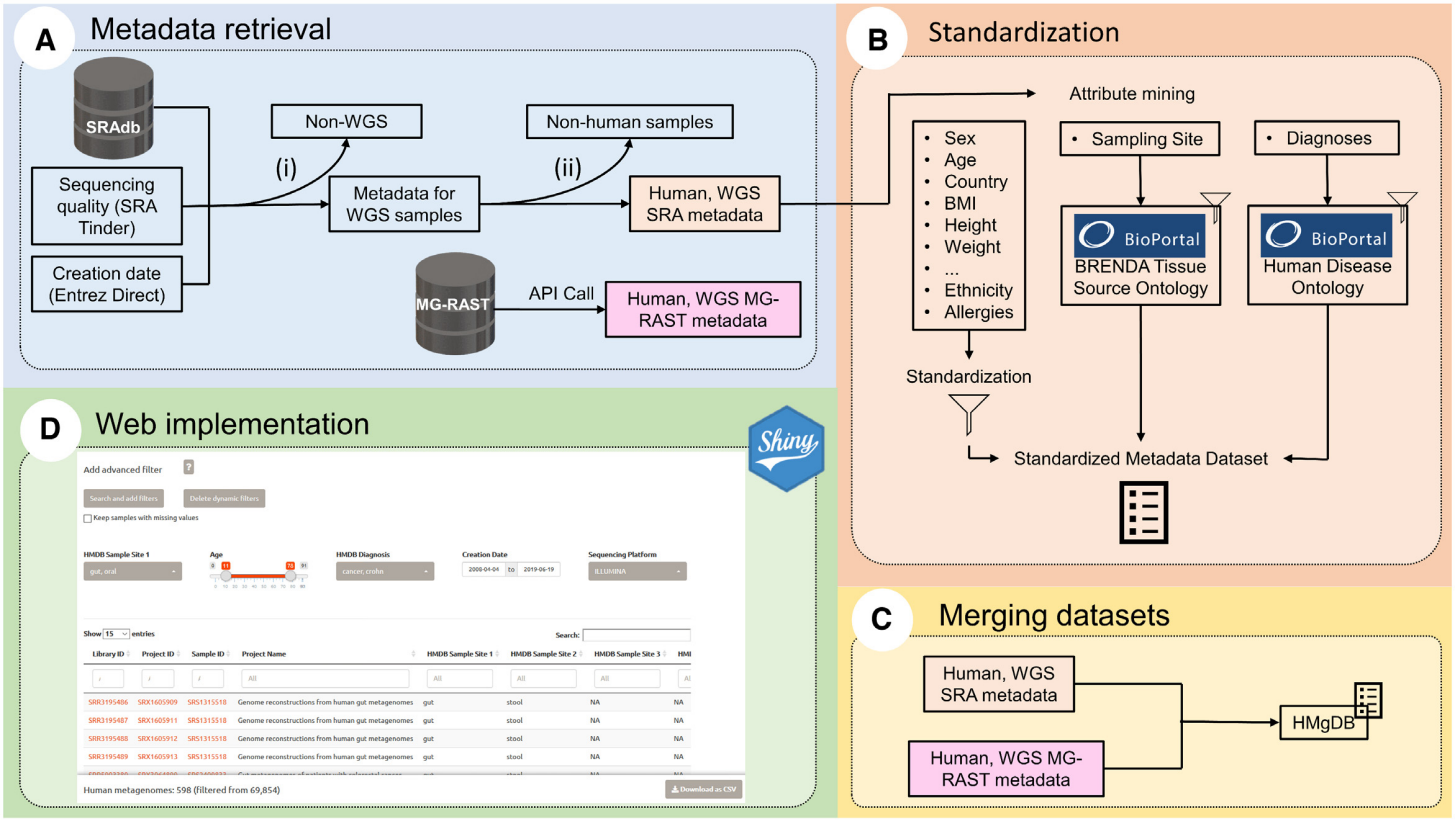
Overview of the HumanMetagenomeDB construction method. (**A**) Parallel metadata retrieval from the SRA and the MG-RAST repositories following two specifics pipelines. For the SRA repository, the removal of non-WGS samples (i) and the removal of non-human samples (ii) were carried out as described in the text. For the MG-RAST repository, the provided API was used to select the samples. (**B**) Attribute mining and Standardization. For the sampling site and diagnoses, the standardization was based on the BioPortal BRENDA Tissue Ontology and the BioPortal Human Disease Ontology, respectively. (**C**) Merging the standardized SRA metadata dataset and MG-RAST metadata dataset. The MG-RAST attributes were adapted to the SRA metadata standard. (**D**) The HumanMetagenomeDB was made available online through a Shiny web implementation. Adapted from (20).

Briefly, we constructed the HumanMetagenomeDB by retrieving the metadata from two source repositories (i.e. SRA or MG-RAST). Samples that were not Whole Genome Sequencing (WGS) data were filtered out. Next, non-human samples were removed. Available attributes were selected, standardized, and the resulting datasets from SRA and MG-RAST were combined. Finally, we implemented a web-application for metadata exploration. An overview of the database construction method is found in Figure 1. Following, we detail the main steps of the database construction.

#### Data retrieval and non-whole genome sequencing removal

For the SRA repository, the first step to retrieve the metadata and remove non-WGS data was to use the PARTIE tool (15). This tool classifies the samples, using a predictive model induced by machine learning, as either WGS or amplicon sequencing, and provides the sample identification runs (i.e. SRA run IDs) of those classified as WGS samples. The list of SRA Runs IDs is located at the PARTIE Github page (https://github.com/linsalrob/partie). After the removal of non-WGS entries, metadata from the remaining metagenomes was retrieved using the SRAdb R package (26), which provides local access to all metadata entries from SRA. With the metadata in hand, new filters were applied to remove the remaining non-WGS samples. Samples with ‘library_strategy’ filled with ‘AMPLICON’ or ‘*RNA*’ were removed. Samples with ‘library_selection’ filled with ‘*PCR*’ were removed, since PCR is generally used as an amplification technique for targeted approaches. Finally, samples with ‘library_source’ different from ‘METAGENOMIC’ or ‘GENOMIC’ were also removed. For the MG-RAST repository, metadata was retrieved using their application program interface (API). To select only WGS samples, we kept samples with the attributes ‘investigation_type’ and ‘seq_meth’ equal to ‘WGS’. Figure 1A summarizes the whole process.

#### Identification of human metagenomes based on the metadata

We used the following strategy to select human metagenomes: (i) we created a dictionary of terms related to the ocean, soil, non-human animals, and human (Supplementary Table S1); (ii) we isolated columns of the metadata table retrieved from the SRA that contained information indicating the origin of the sequence – the selected columns were ‘center_project_name’, ‘study_title’ and ‘sample_attribute’; (iii) each sample was denoted as ‘keep’—if it contained human-related terms –, ‘remove’ – if it contained terms present in the non-human dictionaries—, or ‘missing information’ (hereafter referred to as ‘NA’), resulting in a three-dimensional vector per sample; (iv) if the sample contained only ‘remove’ and ‘NA’, it was removed; (v) if the sample contained only ‘keep’ and ‘NA’, it was kept; (vi) if the sample contained ‘keep’ and ‘remove’ at the same time, it was classified as ‘check’; (vii) and if the sample contained only ‘NA’ it was classified as ‘undefined’; and (viii) all entries that were not labeled as ‘keep’ were removed. After this semi-automatic filter step, we inspected the output manually. Samples containing terms that relate to amplicon sequence, such as 16S, were removed, as well as samples related to transcriptomics (see Supplementary Table S2 for all the terms used to remove non-WGS samples).

#### Standardization of attributes

In SRAdb, all the sample attributes are available in the sample_attribute field. These attributes are written in several different ways, and their values are also non-standardized. Apart from the necessary minimum information, most studies that submitted samples to the SRA database described their metadata differently. To circumvent this aspect, we created a list of synonyms and screened attribute names and their respective values. During data inspection, we identified attributes related to the sample country of origin, coordinates (e.g. latitude and longitude), age, sex, body mass index, ethnicity and several other host characteristics listed in Supplementary Table S3. To standardized the ethnicity attribute we used the definitions created by the U.S. Office of Management and Budget (OMB), and used by the U.S. Census Bureau: White, Black or African American, American Indian or Alaska Native, Asian and Native Hawaiian or Other Pacific Islander (https://www.census.gov/topics/population/race/about.html). The US Census also allows the individual to select more than one option, to state another ethnicity, and to declare to be Hispanic or Not Hispanic. In some cases, the ethnicity information could not be standardized in these categories and was left as stated by the original submitter of the metadata. We also looked for standardization of host exposure attributes, such as diet (e.g. Vegetarian, Omnivore), environmental exposure (e.g. Pets presence, Rural or Urban status), and chemical exposure (e.g. antibiotics use, supplements use). The sampling sites were standardized into eight main categories including gut, oral, skin, ear, lung, vagina, bone, and bio-fluids. Further, we standardized the sample material and sample environment in one column called ‘Sample Site’. The ‘Sample Site’ attribute is further hierarchically structured. We used the BRENDA Tissue and Enzyme Source Ontology (http://bioportal.bioontology.org/ontologies/BTO/?p=classes&conceptid=root) to standardize the ‘Sample Site’ attribute. When possible, diagnosis attributes were also collected and grouped into one single column. Further, when the diagnosis was available, a specific column for each disorder was created, stating whether the sample was positive or negative for each respective disease. To standardize diagnosis, we used the Human Disease Ontology terms (http://bioportal.bioontology.org/ontologies/DOID). We also standardized terms related to control samples, found on Supplementary Table S3. A specific column, called HMgDB_control, was created to describe from which disease study the control sample came from when the information is available on the raw metadata table. Finally, information regarding sample identification, number of sequences, base pair count and sequencing platform were collected directly from the original metadata table. Some attributes and the ontologies used were selected based on the Metagenomics Sequences Sample (MIMS): metagenome/environmental, human-associated; version 5.0 Package (https://www.ncbi.nlm.nih.gov/biosample/docs/packages/MIMS.me.human-associated.5.0/).

Each term used during the standardization of each attribute can be found in Supplementary Table S3. Coordinates were standardized to the format of Decimal Degrees. Dates were standardized to the international standard ISO 8601 (YYYY-MM-DD). Country names were manually labeled based on the ISO 3166-1 standard. Information about the age of individuals was converted to years—rather than days, weeks, or months. Additionally, we recovered quality scores from the SRA samples using SRA-Tinder tool (https://github.com/NCBI-Hackathons/SRA_Tinder), and the creation date of the libraries using the Entrez Direct tool (https://www.ncbi.nlm.nih.gov/books/NBK179288). Finally, we recover the BioProject ID and the PubMed ID using the rentrez tool (https://github.com/ropensci/rentrez). For MG-RAST-retrieved data, selected attributes were mostly already standardized. Therefore, these columns were adapted to the standard created during the standardization of the SRA retrieved metadata. Finally, we calculated the average length of the sequencing read per sample (i.e., basepairs count/sequence count). When the average length of sequencing data generated by Illumina or 454 was larger than 600 bp, we classified the samples as assembled data. The complete set of standardized attributes can be found in Supplementary Table S4. Figure 1B describes the standardization step.

#### Combining SRA and MG-RAST

We manually explored MG-RAST metadata and selected for both equivalent and comparable attributes present in the newly curated SRA metadata (Figure 1C). Equivalent columns names are found in Supplementary Table S5. The standardization of terms found in MG-RAST data was made equivalent to the SRA standardized dataset, and the terms grouped can be found in Supplementary Table S6. The sequencing quality attributes were specific for each original repository. Samples from the SRA uses ‘quality_above_30’ score, and samples from MG-RAST uses ‘drisee_score_raw’.

#### Web app implementation

The HumanMetagenomeDB web-interface was implemented in Shiny (version 1.4.0.2) for R (version 3.6.3). The app was designed with a tab layout. The ‘Home’ tab guides the user through the application. The ‘Quick search’ tab includes the complete dataset with some of the most important filter options. The ‘Advanced search’ tab includes the option to dynamically generate filters for all the available attributes. The ‘Interactive map’ tab includes a way for the user to first select samples according to its geographical location. All the tabs include the feature to visualize the distribution of the selected data. To use it, click on the ‘Visualize’ button. Additionally, a ‘Help’ and a ‘Contact’ tab include information about the attributes and how to understand them. All the packages and their respective versions used to implement the app can be found in the Supplementary File S1. The application is available at https://webapp.ufz.de/hmgdb/. Figure 1D is a screenshot of the webpage containing the dataset.

## RESULTS

### Database content

**Figure 2.**
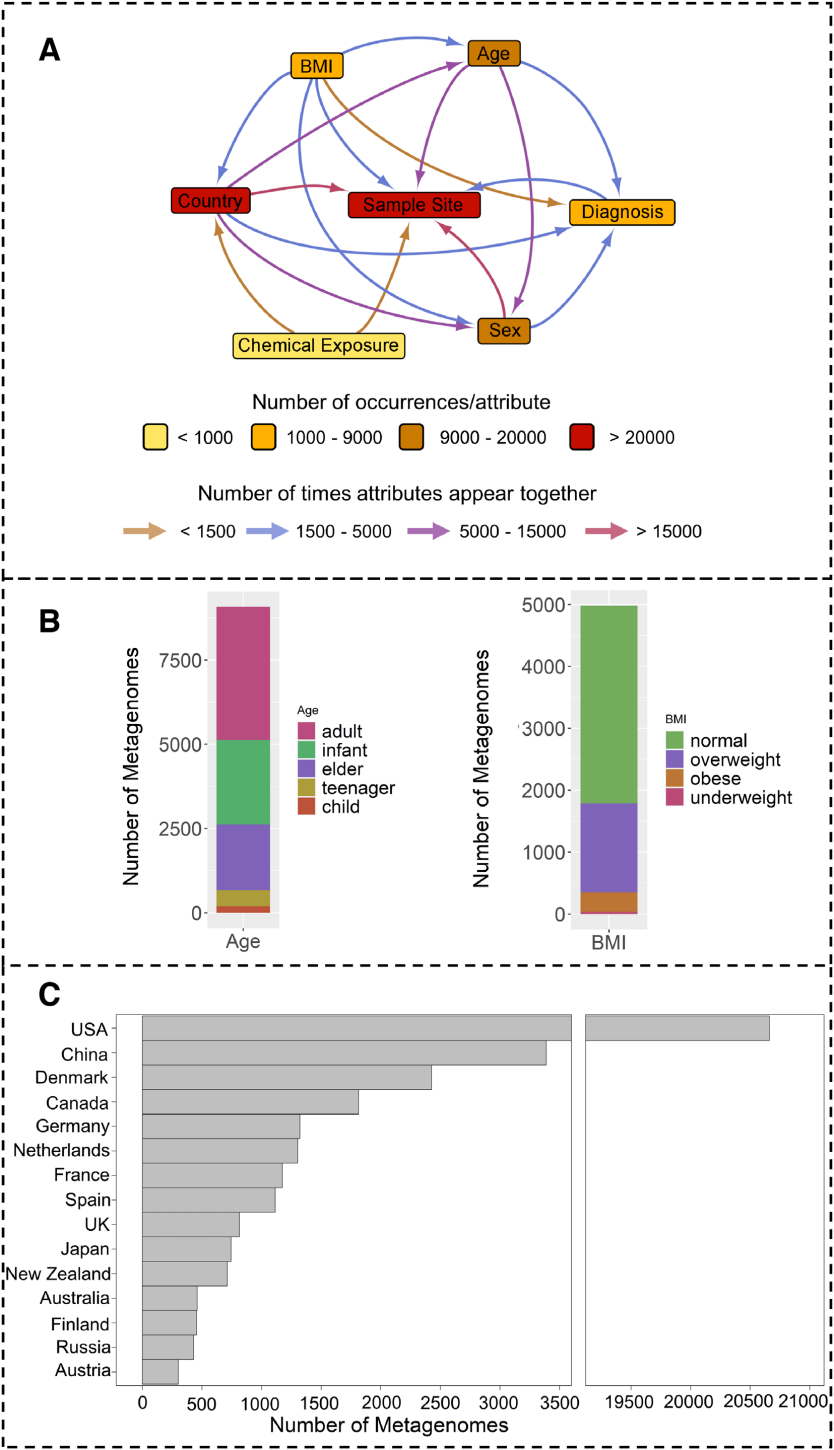
Select descriptions of the HumanMetagenomeDB content. (**A**) A co-occurrence network representing the frequencies of samples containing the information of the attributes in the nodes. The edges indicate the number of samples where the connected attributes appear together. (**B**) A stacked barplot shows the absolute distribution of metagenome samples according to host age (left) and host Body Mass Index (BMI) (right). (**C**) A barplot containing the absolute distribution of metagenomes samples collected in the top 15 countries. The USA barplot scale was adapted. The country contains 20 629 samples and is the most frequent country in the database. For all plots, not assigned values (NA) were omitted.

The HumanMetagenomeDB release 1.0 contains metadata from 69 822 metagenomes from human samples covering 203 meta-attributes. Figure 2A shows a co-occurrence network of some of the most frequent attributes in the HumanMetagenomeDB. The database contains samples from 580 independent studies. Most of the metadata originated from the SRA repository, 95.4% (66 589) since it is increasing faster than MG-RAST. The majority of samples were sequenced using Illumina technology, 94.3% (64 335). Host gender was also annotated, with 47.3% (9 325) of samples derived from females and 52.7% (10 387) from males. Standardized age ranges from just born (0) to 91 years old. To facilitate the search by age, we created an age_class attribute, and the ranges were defined as follows: samples obtained from hosts <3 years old were considered ‘infants’, from 3 to 10 years old as ‘child’, from 10 to 16 as ‘teenager’, from 16 to 50 as ‘adults’, and older than 50 years old samples were classified as ‘elder’. The most abundant age class was adults, representing 43.5% (3 951) of the annotated samples, followed by infants with 27.6% (Figure 2B). The host body mass index of metagenome samples was normally distributed (Gaussian distribution) and ranges from underweight to obese (Figure 2B). Samples from 59 countries were generated, with the United States of America (USA) being the most representative country, with 53.4% of the annotated samples, followed by The People's Republic of China, with 8.7% of the annotated samples (Figure 2C). Concerning the sample site (i.e. body site), 63.5% (27 080) of the annotated samples were gut. The second most abundant sample site was oral, with 19% (8 102) of the samples.

From the disease perspective, HMgDB version 1.0 contains 58 distinct diagnoses. The most abundant disorder documented in the dataset is cancer, corresponding to 20.3% of the samples. To enable meta-analyses between cancer types or relative to non-cancer samples, we subdivided samples obtained from patients with cancer into specific types (e.g. colon cancer). The second most abundant host disorder was Crohn's disease, which is an inflammatory bowel disease. To show relationships between disease types and sampling sites, a co-occurrence network was generated (Supplementary Figure 1). Metagenome samples obtained from the gut or biofluids were more likely to be associated with disease outcomes in the host. This likely stems from the larger number of samples derived from the gut (63.5% or 27 080 samples). Interestingly, while biofluid samples only represent a small fraction of samples (4.3% or 1 838 samples), they are strongly correlated with host disease outcomes indicating that metagenomics analysis of biofluids (e.g., sputum, blood, urine) may represent a powerful diagnostic tool.

Future updates of the HMgDB may encompass more diagnoses information since the lack of clear annotation creates the necessity to manually investigate the original projects. From a nutrition perspective, the HMgDB also contains information regarding the host diet (e.g. Vegetarian, Vegans or Omnivores). As for chemical administration, there are samples from which hosts were using antibiotics, contraceptives, protein supplements, alcoholic drinks, multivitamin supplements, and other more specific chemicals. All these data are captured via searchable ontologies. It is important to notice that some attributes are study-specific, and, therefore, are only present in a small percentage of the total of samples.

### Usage and functionalities

**Figure 3.**
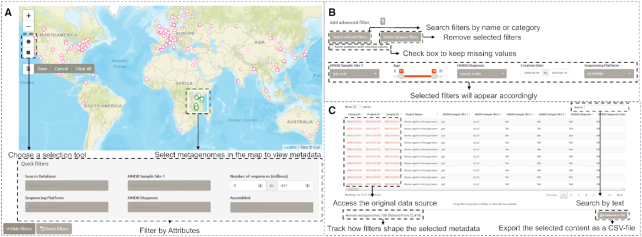
HumanMetagenomeDB user-interface overview. (**A**) The ‘Interactive Map’ tab allows users to select samples according to their geographical location on the world map using a selection tool. (**B**) The ‘Advanced search’ tab allows users the select as much filter as they want. (**C**) Metadata is displayed under the filtering options.

The HumanMetagenomeDB user interface is divided into three main sections. Users can choose the section that best fits their needs. The first section, ‘Quick search’, holds the full content of the databases' current version, as well as the ability to filter the samples by their main characteristics, including Sample Site, Age, and Diagnosis. The ‘Advanced search’ section generates filters for all available attributes of the complete dataset, allowing the user to search for study-specific attributes. Finally, the ‘Interactive Map’ section provides a more intuitive way of selecting metagenomes by regions directly from the world map, although being limited to the metagenomes with a pair of valid geographic coordinates available. The sample identification information per sample—the ‘library id’, ‘project id’, 'sample id’ (when valid), ‘BioProject ID’, and ‘PubMed ID’ (when present)—are hyperlinked to the source databases. All the tabs include the feature to visualize the distribution of the selected data. Under the ‘Visualize’ button the user will be able to see a pie chart pointing the percentage of the data that was selected, an interactive histogram for all the available attributes—which helps the user better understand the distribution of their data—and a summary table for the selected attribute. Figure 3 shows an overview of the HumanMetagenomeDB user-interface.

#### Quick search

The ‘Quick search’ tab allows users to access the full content of HumanMetagenomeDB, as well as to filter the dataset according to the main available attributes. All metagenomes metadata will be shown in the quick search section, including those without valid coordinates. One can filter the entries by using the 30 available main filters or by typing in the search box that is placed at the top of the table. After filtering, metadata of selected entries can be downloaded as a comma-separated values (.csv) file. If the user does not apply any filter, the whole dataset can be downloaded. The steps necessary to obtain underlying raw sequencing data are described next.

#### Advanced search

The ‘Advanced search’ tab generates filters dynamically for all the available attributes of the complete dataset since not all the filters are present in the ‘Quick search’ tab. A checkbox was implemented to allow users to filter out samples with missing values for the chosen attributes. The user can click on the ‘Search and add filters’ button and a window will open. Searches for attributes can be made by name, but they are also organized using the following categories: ‘Sample site’, ‘Host Characteristics’, ‘Diagnosis’, ‘Host Exposure’, ‘Sample location’ and ‘Sequencing Attributes’. After selecting the filters of interest and associated values, one can download the metadata of selected entries as a comma-separated values (.csv) file.

#### Interactive map

The interactive map allows users to identify samples from locations of interest in a world map. Therefore, the Interactive Map contains only samples with valid coordinates. We implemented drawing tools (e.g. polygon or rectangular) to help users to select samples on the map. It is important to note that individual points marked in the map may represent more than one sample since multiple samples can come from the same coordinate position. After the selection of the samples in the map, the selected metagenomes metadata will be shown on the dataset table below the map. The user can further filter the entries by using the same filters present in the ‘Quick search’ tab or by typing in the search box that is placed at the top of the table. After filtering the dataset, the resulted metadata table can be downloaded as a comma-separated values (.csv) file.

#### Downloading the raw data from selected metagenomes

The HumanMetagenomeDB does not store the raw sequence data of the samples. Instead, the application helps scientists to select all relevant samples they would like to analyze. Once the metagenomes are selected, they must be downloaded from their respective repositories. To facilitate the download, we created a python script, called ‘hmgdb_downloader.py’, which uses the CSV file downloaded from the HumanMetagenomeDB to automatically download raw sequences from their repositories. The script is designed for Python3, allowing full interoperability between Linux, Windows, and Mac. The script is provided on the Microbial Data Science group GitHub page (https://github.com/mdsufz/hmgdb_script). Additionally, for Windows users, we created an executable file called ‘hmgdb_downloader.exe’ that can be downloaded directly from the HMgDB page by clicking on the available ‘Download Raw Sequences’ button. The executable can be started in default mode by one click without the need to install Python or any Python module. Both script and executable use SRA ftp download source to download raw data. MG-RAST downloads are performed using https. To better guide the user through the download of the raw sequences, we recorded a series of Youtube video tutorials that can be found on the ‘Help’ page of the HMgDB, or directly under the ‘Download Raw Sequences’ button.

#### Usage example

A researcher interested in searching for differences in the metagenome of humans based on their body mass index and diet type may use the HMgDB to find the samples needed to answer this question. On the Quick search tab under ‘More filters’, the user can search for samples with ‘Host Diet’ information and will find 197 samples from Vegetarians and Omnivores. The user can further select only the samples with BMI ranging from 10.7 to 45.2 and will find 85 samples. After the initial filtering process, the user can click on ‘Visualize’ to explore the selection. One will find that the dataset has 40 female samples and 45 male samples, and most of the samples are from humans between 22 and 31 years old. Finally, the user can download the selected metadata dataset as a CSV file for further analysis and use our provided tool to download the raw sequence data from the selected samples.

#### Database update plan

The number of metagenomic experiments submitted on publicly available repositories, such as the SRA, is continually increasing. To keep the HMgDB updated we plan to perform the addition of the newly submitted samples twice per year: during the first week of February and during the first week of September. One bottleneck that keeps the database to be updated more frequently is the standardization step. This step is semi-automatic and requires manual curation of the terms. The server that maintains the website is going to be continuously supported and new features could be added at any time. If you have any suggestions or requests, please use our Feedback form placed on the Contact tab on the website.

#### Suggestions for good practices

As a major goal of this work was to provide unifying ontologies to facilitate meta-analyses, our database also includes a guide to help scientists to better annotate their metadata when submitting novel metagenome samples. Suggested ontologies can be located under Point 7 in the ‘Help’ tab of the HMgDB website under the title ‘What should I do to include my metagenomes in the HMgDB?’.

## CONCLUSION

The HumanMetagenomeDB centralizes and standardizes metadata for human metagenomes present in the SRA and MG-RAST databases. It covers over 69 822 human-associated metagenomes and 203 attributes. These metagenomes encompass 58 countries, nine main sample sites (body parts), 58 diagnoses, age ranging from just born to 91 years old, and body mass index ranging from underweight to obese. Further, our novel database has a user-friendly interface that allows users to explore, select, and download curated metadata, helping scientists from different domain areas to select samples according to their interests. Finally, the availability of our database lays the groundwork for a unified human metagenomic database, providing a simple guide for the harmonization of new metadata and ontologies. The HMgDB is in its release 1.0, and due to the exponential number of novel experiments added to public repositories, it will be updated twice a year. Taken together, our database improves harmonization of metadata ontologies of human metagenome and facilitates simple querying, interpretation, and simple access to underlying data across distinct studies.

## DATA AVAILABILITY

The HumanMetagenomeDB is available at https://webapp.ufz.de/hmgdb/.

## Supplementary Material

gkaa1031_Supplemental_Files

## Notes

Present address: Ulisses Rocha, Department of Environmental Microbiology, Helmholtz Centre for Environmental Research – UFZ GmbH, Leipzig, Saxony 04318, Leipzig.
