# Supplementary material for: HumanMetagenomeDB: a public repository of curated and standardized metadata for human metagenomes
Source: Nucleic Acids Res. 2020 Nov 22;49(D1):D743–50. doi: 10.1093/nar/gkaa1031 (PMC7778935; doi:10.1093/nar/gkaa1031)
Supplement: gkaa1031_Supplemental_Files [file gkaa1031_supplemental_files.zip › Kasmanas_HMgDB_NAR_supp_file_S1_r_package_versions.docx]

**Supplementary File S1**. R packages and the respective versions used during the implementation of the HumanMetagenomeDB Web-Application.

{

"R": {

"Version": "3.6.3",

"Repositories": [

{

"Name": "CRAN",

"URL": "https://cran.rstudio.com"

}

]

},

"Packages": {

"BH": {

"Package": "BH",

"Version": "1.72.0-3",

"Source": "Repository",

"Repository": "CRAN",

"Hash": "8f9ce74c6417d61f0782cbae5fd2b7b0"

},

"DT": {

"Package": "DT",

"Version": "0.15",

"Source": "Repository",

"Repository": "CRAN",

"Hash": "85738c69035e67ec4b484a5e02640ef6"

},

"MASS": {

"Package": "MASS",

"Version": "7.3-51.5",

"Source": "Repository",

"Repository": "CRAN",

"Hash": "9efe80472b21189ebab1b74169808c26"

},

"Matrix": {

"Package": "Matrix",

"Version": "1.2-18",

"Source": "Repository",

"Repository": "CRAN",

"Hash": "08588806cba69f04797dab50627428ed"

},

"R6": {

"Package": "R6",

"Version": "2.4.1",

"Source": "Repository",

"Repository": "CRAN",

"Hash": "292b54f8f4b94669b08f94e5acce6be2"

},

"RColorBrewer": {

"Package": "RColorBrewer",

"Version": "1.1-2",

"Source": "Repository",

"Repository": "CRAN",

"Hash": "e031418365a7f7a766181ab5a41a5716"

},

"Rcpp": {

"Package": "Rcpp",

"Version": "1.0.5",

"Source": "Repository",

"Repository": "CRAN",

"Hash": "125dc7a0ed375eb68c0ce533b48d291f"

},

"askpass": {

"Package": "askpass",

"Version": "1.1",

"Source": "Repository",

"Repository": "CRAN",

"Hash": "e8a22846fff485f0be3770c2da758713"

},

"assertthat": {

"Package": "assertthat",

"Version": "0.2.1",

"Source": "Repository",

"Repository": "CRAN",

"Hash": "50c838a310445e954bc13f26f26a6ecf"

},

"backports": {

"Package": "backports",

"Version": "1.1.9",

"Source": "Repository",

"Repository": "CRAN",

"Hash": "b29e2d989dfb2e71ca3fd7d5bb1c0d58"

},

"base64enc": {

"Package": "base64enc",

"Version": "0.1-3",

"Source": "Repository",

"Repository": "CRAN",

"Hash": "543776ae6848fde2f48ff3816d0628bc"

},

"brew": {

"Package": "brew",

"Version": "1.0-6",

"Source": "Repository",

"Repository": "CRAN",

"Hash": "92a5f887f9ae3035ac7afde22ba73ee9"

},

"callr": {

"Package": "callr",

"Version": "3.4.4",

"Source": "Repository",

"Repository": "CRAN",

"Hash": "e56fe17ffeddfdcfcef40981e41e1c40"

},

"cli": {

"Package": "cli",

"Version": "2.0.2",

"Source": "Repository",

"Repository": "CRAN",

"Hash": "ff0becff7bfdfe3f75d29aff8f3172dd"

},

"clipr": {

"Package": "clipr",

"Version": "0.7.0",

"Source": "Repository",

"Repository": "CRAN",

"Hash": "08cf4045c149a0f0eaf405324c7495bd"

},

"colorspace": {

"Package": "colorspace",

"Version": "1.4-1",

"Source": "Repository",

"Repository": "CRAN",

"Hash": "6b436e95723d1f0e861224dd9b094dfb"

},

"commonmark": {

"Package": "commonmark",

"Version": "1.7",

"Source": "Repository",

"Repository": "CRAN",

"Hash": "0f22be39ec1d141fd03683c06f3a6e67"

},

"covr": {

"Package": "covr",

"Version": "3.5.0",

"Source": "Repository",

"Repository": "CRAN",

"Hash": "cbc6df1ef6ee576f844f973c1fc04ab4"

},

"cpp11": {

"Package": "cpp11",

"Version": "0.2.1",

"Source": "Repository",

"Repository": "CRAN",

"Hash": "c4c83167f43ca762a9fa998d4fead3ae"

},

"crayon": {

"Package": "crayon",

"Version": "1.3.4",

"Source": "Repository",

"Repository": "CRAN",

"Hash": "0d57bc8e27b7ba9e45dba825ebc0de6b"

},

"crosstalk": {

"Package": "crosstalk",

"Version": "1.1.0.1",

"Source": "Repository",

"Repository": "CRAN",

"Hash": "ae55f5d7c02f0ab43c58dd050694f2b4"

},

"curl": {

"Package": "curl",

"Version": "4.3",

"Source": "Repository",

"Repository": "CRAN",

"Hash": "2b7d10581cc730804e9ed178c8374bd6"

},

"data.table": {

"Package": "data.table",

"Version": "1.13.0",

"Source": "Repository",

"Repository": "CRAN",

"Hash": "06d5863292e2e8ffbb063ce34e77bb2a"

},

"desc": {

"Package": "desc",

"Version": "1.2.0",

"Source": "Repository",

"Repository": "CRAN",

"Hash": "6c8fe8fa26a23b79949375d372c7b395"

},

"devtools": {

"Package": "devtools",

"Version": "2.3.1",

"Source": "Repository",

"Repository": "CRAN",

"Hash": "271df6a328617c64149283e98b1cd8da"

},

"digest": {

"Package": "digest",

"Version": "0.6.25",

"Source": "Repository",

"Repository": "CRAN",

"Hash": "f697db7d92b7028c4b3436e9603fb636"

},

"dplyr": {

"Package": "dplyr",

"Version": "1.0.2",

"Source": "Repository",

"Repository": "CRAN",

"Hash": "d0509913b27ea898189ee664b6030dc2"

},

"ellipsis": {

"Package": "ellipsis",

"Version": "0.3.1",

"Source": "Repository",

"Repository": "CRAN",

"Hash": "fd2844b3a43ae2d27e70ece2df1b4e2a"

},

"evaluate": {

"Package": "evaluate",

"Version": "0.14",

"Source": "Repository",

"Repository": "CRAN",

"Hash": "ec8ca05cffcc70569eaaad8469d2a3a7"

},

"fansi": {

"Package": "fansi",

"Version": "0.4.1",

"Source": "Repository",

"Repository": "CRAN",

"Hash": "7fce217eaaf8016e72065e85c73027b5"

},

"farver": {

"Package": "farver",

"Version": "2.0.3",

"Source": "Repository",

"Repository": "CRAN",

"Hash": "dad6793a5a1f73c8e91f1a1e3e834b05"

},

"fastmap": {

"Package": "fastmap",

"Version": "1.0.1",

"Source": "Repository",

"Repository": "CRAN",

"Hash": "83ab58a0518afe3d17e41da01af13b60"

},

"foreign": {

"Package": "foreign",

"Version": "0.8-75",

"Source": "Repository",

"Repository": "CRAN",

"Hash": "9a7efaa7320b9f327a904d1e615b0b46"

},

"fs": {

"Package": "fs",

"Version": "1.5.0",

"Source": "Repository",

"Repository": "CRAN",

"Hash": "44594a07a42e5f91fac9f93fda6d0109"

},

"generics": {

"Package": "generics",

"Version": "0.0.2",

"Source": "Repository",

"Repository": "CRAN",

"Hash": "b8cff1d1391fd1ad8b65877f4c7f2e53"

},

"geoshaper": {

"Package": "geoshaper",

"Version": "0.1.0",

"Source": "GitHub",

"RemoteType": "github",

"RemoteHost": "api.github.com",

"RemoteRepo": "geoshaper",

"RemoteUsername": "RedOakStrategic",

"RemoteRef": "HEAD",

"RemoteSha": "b3fd1b677cc5208edbecc21952b4eba7121c7803",

"Hash": "36e6dd53ad62989a78423bf0d152ac9d"

},

"ggplot2": {

"Package": "ggplot2",

"Version": "3.3.2",

"Source": "Repository",

"Repository": "CRAN",

"Hash": "4ded8b439797f7b1693bd3d238d0106b"

},

"gh": {

"Package": "gh",

"Version": "1.1.0",

"Source": "Repository",

"Repository": "CRAN",

"Hash": "89ea5998938d1ad55f035c8a86f96b74"

},

"git2r": {

"Package": "git2r",

"Version": "0.27.1",

"Source": "Repository",

"Repository": "CRAN",

"Hash": "531a82d1beed1f545beb25f4f5945bf7"

},

"glue": {

"Package": "glue",

"Version": "1.4.2",

"Source": "Repository",

"Repository": "CRAN",

"Hash": "6efd734b14c6471cfe443345f3e35e29"

},

"gridExtra": {

"Package": "gridExtra",

"Version": "2.3",

"Source": "Repository",

"Repository": "CRAN",

"Hash": "7d7f283939f563670a697165b2cf5560"

},

"gtable": {

"Package": "gtable",

"Version": "0.3.0",

"Source": "Repository",

"Repository": "CRAN",

"Hash": "ac5c6baf7822ce8732b343f14c072c4d"

},

"hexbin": {

"Package": "hexbin",

"Version": "1.28.1",

"Source": "Repository",

"Repository": "CRAN",

"Hash": "3d59212f2814d65dff517e6899813c58"

},

"highr": {

"Package": "highr",

"Version": "0.8",

"Source": "Repository",

"Repository": "CRAN",

"Hash": "4dc5bb88961e347a0f4d8aad597cbfac"

},

"htmltools": {

"Package": "htmltools",

"Version": "0.5.0",

"Source": "Repository",

"Repository": "CRAN",

"Hash": "7d651b7131794fe007b1ad6f21aaa401"

},

"htmlwidgets": {

"Package": "htmlwidgets",

"Version": "1.5.1",

"Source": "Repository",

"Repository": "CRAN",

"Hash": "41bace23583fbc25089edae324de2dc3"

},

"httpuv": {

"Package": "httpuv",

"Version": "1.5.4",

"Source": "Repository",

"Repository": "CRAN",

"Hash": "4e6dabb220b006ccdc3b3b5ff993b205"

},

"httr": {

"Package": "httr",

"Version": "1.4.2",

"Source": "Repository",

"Repository": "CRAN",

"Hash": "a525aba14184fec243f9eaec62fbed43"

},

"ini": {

"Package": "ini",

"Version": "0.3.1",

"Source": "Repository",

"Repository": "CRAN",

"Hash": "6154ec2223172bce8162d4153cda21f7"

},

"isoband": {

"Package": "isoband",

"Version": "0.2.2",

"Source": "Repository",

"Repository": "CRAN",

"Hash": "6e58bd3d6b3dd82a944cd6f05ade228f"

},

"jsonlite": {

"Package": "jsonlite",

"Version": "1.7.0",

"Source": "Repository",

"Repository": "CRAN",

"Hash": "2657f20b9a74c996c602e74ebe540b06"

},

"knitr": {

"Package": "knitr",

"Version": "1.29",

"Source": "Repository",

"Repository": "CRAN",

"Hash": "e5f4c41c17df8cdf7b0df12117c0d99a"

},

"labeling": {

"Package": "labeling",

"Version": "0.3",

"Source": "Repository",

"Repository": "CRAN",

"Hash": "73832978c1de350df58108c745ed0e3e"

},

"later": {

"Package": "later",

"Version": "1.1.0.1",

"Source": "Repository",

"Repository": "CRAN",

"Hash": "d0a62b247165aabf397fded504660d8a"

},

"lattice": {

"Package": "lattice",

"Version": "0.20-38",

"Source": "Repository",

"Repository": "CRAN",

"Hash": "848f8c593fd1050371042d18d152e3d7"

},

"lazyeval": {

"Package": "lazyeval",

"Version": "0.2.2",

"Source": "Repository",

"Repository": "CRAN",

"Hash": "d908914ae53b04d4c0c0fd72ecc35370"

},

"leaflet": {

"Package": "leaflet",

"Version": "2.0.3",

"Source": "Repository",

"Repository": "CRAN",

"Hash": "3f3f5e5603fc791dfb77279ad84cc711"

},

"leaflet.extras": {

"Package": "leaflet.extras",

"Version": "1.0.0",

"Source": "Repository",

"Repository": "CRAN",

"Hash": "8dbfc2c4d7ca2660971caf1153ca95c2"

},

"leaflet.providers": {

"Package": "leaflet.providers",

"Version": "1.9.0",

"Source": "Repository",

"Repository": "CRAN",

"Hash": "d3082a7beac4a1aeb96100ff06265d7e"

},

"lifecycle": {

"Package": "lifecycle",

"Version": "0.2.0",

"Source": "Repository",

"Repository": "CRAN",

"Hash": "361811f31f71f8a617a9a68bf63f1f42"

},

"magrittr": {

"Package": "magrittr",

"Version": "1.5",

"Source": "Repository",

"Repository": "CRAN",

"Hash": "1bb58822a20301cee84a41678e25d9b7"

},

"maptools": {

"Package": "maptools",

"Version": "1.0-2",

"Source": "Repository",

"Repository": "CRAN",

"Hash": "1cb5cb7bbab76318944e3794ff2512ae"

},

"markdown": {

"Package": "markdown",

"Version": "1.1",

"Source": "Repository",

"Repository": "CRAN",

"Hash": "61e4a10781dd00d7d81dd06ca9b94e95"

},

"memoise": {

"Package": "memoise",

"Version": "1.1.0",

"Source": "Repository",

"Repository": "CRAN",

"Hash": "58baa74e4603fcfb9a94401c58c8f9b1"

},

"mgcv": {

"Package": "mgcv",

"Version": "1.8-31",

"Source": "Repository",

"Repository": "CRAN",

"Hash": "4bb7e0c4f3557583e1e8d3c9ffb8ba5c"

},

"mime": {

"Package": "mime",

"Version": "0.9",

"Source": "Repository",

"Repository": "CRAN",

"Hash": "e87a35ec73b157552814869f45a63aa3"

},

"munsell": {

"Package": "munsell",

"Version": "0.5.0",

"Source": "Repository",

"Repository": "CRAN",

"Hash": "6dfe8bf774944bd5595785e3229d8771"

},

"nlme": {

"Package": "nlme",

"Version": "3.1-144",

"Source": "Repository",

"Repository": "CRAN",

"Hash": "e80d41932d3cc235ccbbbb9732ae162e"

},

"openssl": {

"Package": "openssl",

"Version": "1.4.2",

"Source": "Repository",

"Repository": "CRAN",

"Hash": "b3209c62052922b6c629544d94c8fa8a"

},

"pillar": {

"Package": "pillar",

"Version": "1.4.6",

"Source": "Repository",

"Repository": "CRAN",

"Hash": "bdf26e55ccb7df3e49a490150277f002"

},

"pkgbuild": {

"Package": "pkgbuild",

"Version": "1.1.0",

"Source": "Repository",

"Repository": "CRAN",

"Hash": "404684bc4e3685007f9720adf13b06c1"

},

"pkgconfig": {

"Package": "pkgconfig",

"Version": "2.0.3",

"Source": "Repository",

"Repository": "CRAN",

"Hash": "01f28d4278f15c76cddbea05899c5d6f"

},

"pkgload": {

"Package": "pkgload",

"Version": "1.1.0",

"Source": "Repository",

"Repository": "CRAN",

"Hash": "b6b150cd4709e0c0c9b5d51ac4376282"

},

"plotly": {

"Package": "plotly",

"Version": "4.9.2.1",

"Source": "Repository",

"Repository": "CRAN",

"Hash": "b08edf378e0e38959a6983e3d5902795"

},

"png": {

"Package": "png",

"Version": "0.1-7",

"Source": "Repository",

"Repository": "CRAN",

"Hash": "03b7076c234cb3331288919983326c55"

},

"praise": {

"Package": "praise",

"Version": "1.0.0",

"Source": "Repository",

"Repository": "CRAN",

"Hash": "a555924add98c99d2f411e37e7d25e9f"

},

"prettyunits": {

"Package": "prettyunits",

"Version": "1.1.1",

"Source": "Repository",

"Repository": "CRAN",

"Hash": "95ef9167b75dde9d2ccc3c7528393e7e"

},

"processx": {

"Package": "processx",

"Version": "3.4.4",

"Source": "Repository",

"Repository": "CRAN",

"Hash": "03446ed0b8129916f73676726cb3c48f"

},

"promises": {

"Package": "promises",

"Version": "1.1.1",

"Source": "Repository",

"Repository": "CRAN",

"Hash": "a8730dcbdd19f9047774909f0ec214a4"

},

"ps": {

"Package": "ps",

"Version": "1.3.4",

"Source": "Repository",

"Repository": "CRAN",

"Hash": "a54a7dfd68124abb2225dbfa9a85c457"

},

"purrr": {

"Package": "purrr",

"Version": "0.3.4",

"Source": "Repository",

"Repository": "CRAN",

"Hash": "97def703420c8ab10d8f0e6c72101e02"

},

"raster": {

"Package": "raster",

"Version": "3.3-13",

"Source": "Repository",

"Repository": "CRAN",

"Hash": "51cfecf7b9518d46b119f92d581616b6"

},

"rcmdcheck": {

"Package": "rcmdcheck",

"Version": "1.3.3",

"Source": "Repository",

"Repository": "CRAN",

"Hash": "ed95895886dab6d2a584da45503555da"

},

"rematch2": {

"Package": "rematch2",

"Version": "2.1.2",

"Source": "Repository",

"Repository": "CRAN",

"Hash": "76c9e04c712a05848ae7a23d2f170a40"

},

"remotes": {

"Package": "remotes",

"Version": "2.2.0",

"Source": "Repository",

"Repository": "CRAN",

"Hash": "430a0908aee75b1fcba0e62857cab0ce"

},

"renv": {

"Package": "renv",

"Version": "0.12.0",

"Source": "Repository",

"Repository": "CRAN",

"Hash": "7340c71f46a0fd16506cfa804e224e44"

},

"rex": {

"Package": "rex",

"Version": "1.2.0",

"Source": "Repository",

"Repository": "CRAN",

"Hash": "093584b944440c5cd07a696b3c8e0e4c"

},

"rlang": {

"Package": "rlang",

"Version": "0.4.7",

"Source": "Repository",

"Repository": "CRAN",

"Hash": "c06d2a6887f4b414f8e927afd9ee976a"

},

"rmarkdown": {

"Package": "rmarkdown",

"Version": "2.3",

"Source": "Repository",

"Repository": "CRAN",

"Hash": "202260e1b2c410edc086d5b8f1ed946e"

},

"roxygen2": {

"Package": "roxygen2",

"Version": "7.1.1",

"Source": "Repository",

"Repository": "CRAN",

"Hash": "fcd94e00cc409b25d07ca50f7bf339f5"

},

"rprojroot": {

"Package": "rprojroot",

"Version": "1.3-2",

"Source": "Repository",

"Repository": "CRAN",

"Hash": "f6a407ae5dd21f6f80a6708bbb6eb3ae"

},

"rstudioapi": {

"Package": "rstudioapi",

"Version": "0.11",

"Source": "Repository",

"Repository": "CRAN",

"Hash": "33a5b27a03da82ac4b1d43268f80088a"

},

"rversions": {

"Package": "rversions",

"Version": "2.0.2",

"Source": "Repository",

"Repository": "CRAN",

"Hash": "0ec41191f744d0f5afad8c6f35cc36e4"

},

"scales": {

"Package": "scales",

"Version": "1.1.1",

"Source": "Repository",

"Repository": "CRAN",

"Hash": "6f76f71042411426ec8df6c54f34e6dd"

},

"sessioninfo": {

"Package": "sessioninfo",

"Version": "1.1.1",

"Source": "Repository",

"Repository": "CRAN",

"Hash": "308013098befe37484df72c39cf90d6e"

},

"shiny": {

"Package": "shiny",

"Version": "1.5.0",

"Source": "Repository",

"Repository": "CRAN",

"Hash": "ee4ed72d7a5047d9e73cf922ad66e9c9"

},

"shinyBS": {

"Package": "shinyBS",

"Version": "0.61",

"Source": "Repository",

"Repository": "CRAN",

"Hash": "f895dafd39733c4a70d425f605a832e7"

},

"shinyWidgets": {

"Package": "shinyWidgets",

"Version": "0.5.3",

"Source": "Repository",

"Repository": "CRAN",

"Hash": "9b31c168255d8f0b99fc434b58f1ccba"

},

"shinyjs": {

"Package": "shinyjs",

"Version": "2.0.0",

"Source": "Repository",

"Repository": "CRAN",

"Hash": "9ddfc91d4280eaa34c2103951538976f"

},

"shinythemes": {

"Package": "shinythemes",

"Version": "1.1.2",

"Source": "Repository",

"Repository": "CRAN",

"Hash": "8f047210d7d68ea4860a3c0d8cced272"

},

"sourcetools": {

"Package": "sourcetools",

"Version": "0.1.7",

"Source": "Repository",

"Repository": "CRAN",

"Hash": "947e4e02a79effa5d512473e10f41797"

},

"sp": {

"Package": "sp",

"Version": "1.4-2",

"Source": "Repository",

"Repository": "CRAN",

"Hash": "3290eebc34ba4df5e213878d54c1e623"

},

"stringi": {

"Package": "stringi",

"Version": "1.5.3",

"Source": "Repository",

"Repository": "CRAN",

"Hash": "a063ebea753c92910a4cca7b18bc1f05"

},

"stringr": {

"Package": "stringr",

"Version": "1.4.0",

"Source": "Repository",

"Repository": "CRAN",

"Hash": "0759e6b6c0957edb1311028a49a35e76"

},

"sys": {

"Package": "sys",

"Version": "3.4",

"Source": "Repository",

"Repository": "CRAN",

"Hash": "b227d13e29222b4574486cfcbde077fa"

},

"testthat": {

"Package": "testthat",

"Version": "2.3.2",

"Source": "Repository",

"Repository": "CRAN",

"Hash": "0829b987b8961fb07f3b1b64a2fbc495"

},

"tibble": {

"Package": "tibble",

"Version": "3.0.3",

"Source": "Repository",

"Repository": "CRAN",

"Hash": "08bd36bd34b20d4f7971d49e81deaab0"

},

"tidyr": {

"Package": "tidyr",

"Version": "1.1.2",

"Source": "Repository",

"Repository": "CRAN",

"Hash": "c40b2d5824d829190f4b825f4496dfae"

},

"tidyselect": {

"Package": "tidyselect",

"Version": "1.1.0",

"Source": "Repository",

"Repository": "CRAN",

"Hash": "6ea435c354e8448819627cf686f66e0a"

},

"tinytex": {

"Package": "tinytex",

"Version": "0.25",

"Source": "Repository",

"Repository": "CRAN",

"Hash": "a4b9662282097d1033c60420dcb83350"

},

"usethis": {

"Package": "usethis",

"Version": "1.6.1",

"Source": "Repository",

"Repository": "CRAN",

"Hash": "e1985f5a9985fea2e338fa7eb99018ca"

},

"utf8": {

"Package": "utf8",

"Version": "1.1.4",

"Source": "Repository",

"Repository": "CRAN",

"Hash": "4a5081acfb7b81a572e4384a7aaf2af1"

},

"vctrs": {

"Package": "vctrs",

"Version": "0.3.4",

"Source": "Repository",

"Repository": "CRAN",

"Hash": "0bc90078aeee42f2520b1d0a33bd6758"

},

"viridis": {

"Package": "viridis",

"Version": "0.5.1",

"Source": "Repository",

"Repository": "CRAN",

"Hash": "6f6b49e5b3b5ee5a6d0c28bf1b4b9eb3"

},

"viridisLite": {

"Package": "viridisLite",

"Version": "0.3.0",

"Source": "Repository",

"Repository": "CRAN",

"Hash": "ce4f6271baa94776db692f1cb2055bee"

},

"waiter": {

"Package": "waiter",

"Version": "0.1.2",

"Source": "Repository",

"Repository": "CRAN",

"Hash": "040479b66f4ed83653ccb8d136f221a6"

},

"whisker": {

"Package": "whisker",

"Version": "0.4",

"Source": "Repository",

"Repository": "CRAN",

"Hash": "ca970b96d894e90397ed20637a0c1bbe"

},

"withr": {

"Package": "withr",

"Version": "2.2.0",

"Source": "Repository",

"Repository": "CRAN",

"Hash": "ecd17882a0b4419545691e095b74ee89"

},

"xfun": {

"Package": "xfun",

"Version": "0.17",

"Source": "Repository",

"Repository": "CRAN",

"Hash": "5016afba2a931ac92bbb3c6feb03d2c8"

},

"xml2": {

"Package": "xml2",

"Version": "1.3.2",

"Source": "Repository",

"Repository": "CRAN",

"Hash": "d4d71a75dd3ea9eb5fa28cc21f9585e2"

},

"xopen": {

"Package": "xopen",

"Version": "1.0.0",

"Source": "Repository",

"Repository": "CRAN",

"Hash": "6c85f015dee9cc7710ddd20f86881f58"

},

"xtable": {

"Package": "xtable",

"Version": "1.8-4",

"Source": "Repository",

"Repository": "CRAN",

"Hash": "b8acdf8af494d9ec19ccb2481a9b11c2"

},

"yaml": {

"Package": "yaml",

"Version": "2.2.1",

"Source": "Repository",

"Repository": "CRAN",

"Hash": "2826c5d9efb0a88f657c7a679c7106db"

}

}

}
