# Supplementary figures and images for: HumanMetagenomeDB: a public repository of curated and standardized metadata for human metagenomes
Source: Nucleic Acids Res. 2020 Nov 22;49(D1):D743–50. doi: 10.1093/nar/gkaa1031 (PMC7778935; doi:10.1093/nar/gkaa1031)

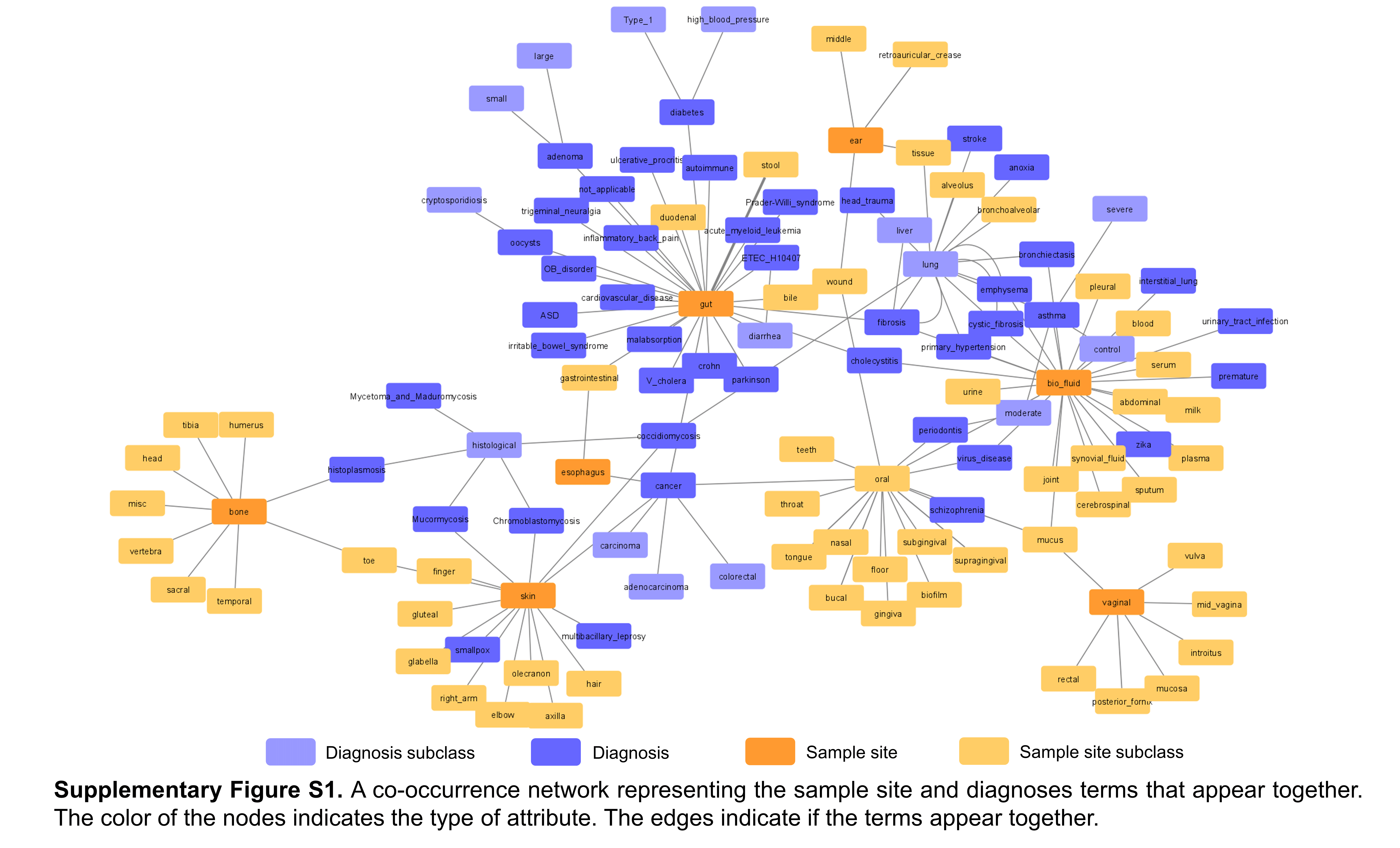

Supplement: gkaa1031_Supplemental_Files [file gkaa1031_supplemental_files.zip › Kasmanas_HMgDB_NAR_Supplementary_figure_S1_300.png]
